# Recherches Anatomiques, Pathologiques Et Thérapeutiques, Sur La Maladie Connue Sous Les Noms De Fièvre Typhoïde, &c.

**Published:** 1841-02-27

**Authors:** 


### BIBLIOGRAPHICAL NOTICE.

*Recherches Anatomiques, Pathologiques et Thérapeutiques, sur la maladie connue sous les noms de Fièvre Typhoïde, &c.* Par P. C. A. Louis. Deuxiéme Edition, considérablement augmentée. A Paris: 1841.

*Remarks, Anatomical, Pathological, and Therapeutical, on the disease known under the name of Typhoid Fever, &c., compared with the most frequent acute diseases.* By P. C. A. Louis, Physician to the Hôtel Dieu, Perpetual President of the Society of Observation, &c. Second Edition, considerably increased. 2 vols., pp. 542–523. Paris, 1841.

Few works published of late years have attracted more notice from the medical profession than that of Dr. Louis on typhoid fever. It is the most elaborate and interesting of his productions, and developes more fully than any other the peculiar method of research pursued by its author. The typhoid fever of Paris was examined with a double object; first, to ascertain what were the symptoms and pathological characters of the disease, or to define what it was; secondly, to point out the distinction between it and simple gastro-enteritis, as it was termed by M. Broussais. The latter object is now of little interest; few care for the demonstrative proof that typhoid fever is not a mere gastro-enteritis; the fact is demonstrated and it is admitted that it is something else,—but, notwithstanding the invaluable labours of Dr. Louis, there are many men of more or less eminence in the profession who are not satisfied of the accuracy of his results. As these results are professedly demonstrative, it is very clear that there is either a defect in the chain of reasoning pursued by the author, or that the readers are incapable of appreciating the rules of accurate induction when applied to medicine.

So far as the direct conclusions of Dr. Louis extend, there is no doubt of their correctness; and if the convictions of the reader are imperfect, the fault rests with him, and not with the author. But there is a wide difference between conclusions and opinions; and notwithstanding the aversion entertained by Dr. Louis for speculative notions, he has not been able to avoid them completely, for demonstration is limited; and where rigorous proof does not reach, the author is either reduced to silence, or is compelled to announce a belief which may or may not be well founded. When opinions are formed by an accurate observer, they are not necessarily more correct than those of a visionary theorist: in some instances they may be less so, for the turn of mind and habit of induction, which are best adapted for an observer, are often unsuitable for abstract reasoning, and there is a sort of discrepancy between the probable opinions and the positive conclusions.

Thus, it is demonstrated by Dr. Louis, that peculiar lesions of the intestinal follicles take place in typhoid fever,—and it is supposed by him, though with some reservation, that this lesion is the cause of the symptoms. The first part of this sentence is correct, and is well established; the second is doubtful, and probably erroneous. In the present edition of the work the error is corrected, and the lesion of the follicles is considered merely as the anatomical character of the disorder. In the chapter containing the summary of the pathological lesions, the relative frequency of the various alterations is perfectly well established, and it is proven that those only connected with the follicles of the ileum and cœcum are at all fixed; but at the conclusion of the chapter we find a statement of a different nature. That is, a strong doubt as to the utility of revulsives, because a primary inflammation will excite numerous others of a secondary nature; evidently in allusion to the secondary inflammations of typhoid fever which seem to follow the lesion of the glands of Peyer. But how many modifying circumstances are there in inflammation, and how can we infer that a specific inflammation can be modified like an ordinary one, by revulsives; and if it cannot be, should we conclude that the laws of revulsion are false.

It is these opinions of Dr. Louis which are right or wrong, like those of other writers; that have, to a certain extent, withdrawn the attention of conscientious physicians from his excellent and well-sustained results, and at the same time have furnished a point of attack for the ill-natured critics, who have neither the power to appreciate the beauty of rigid observation in medicine, nor the desire to admit results which are not only at variance with, but destroy their own vague notions. This is not a just tribute to one who has rendered the most signal services to medicine, not only by attaching a new importance to statistics, which we do not consider his greatest merit, but by applying a thorough system of analysis and observation to the study of disease. Not that observation was before unknown or disregarded, but a change had taken place in the mode; the good old-fashioned way had, to a certain extent, fallen into disuse, and for a time no new system of observing had succeeded to that which was, to a great degree, neglected. The minds of men required separation and analysis of parts before a new synthesis could be attempted; and this new method was certainly furnished by Dr. Louis. To carry this plan into effect, required a power of mind, a concentration of thought, and a conscientious attachment to truth, which belong to few individuals; something more than a strong inductive faculty was necessary; there must be enthusiasm for the science, and a perseverance in the pursuit of it, which could carry the author towards his object at the sacrifice of convenience, and at the cost of an exclusive devotion of six or seven years of life.

The results of this labour are contained mainly in the treatises on typhoid fever, phthisis, and a volume of essays. Since the publication of these works, Dr. Louis has written the work on yellow fever, printed at Boston in an English translation by Dr. Shattuck; a monograph on blood-letting, a reply to the Examen of Dr. Broussais, and a number of memoirs on various subjects.

The second edition of the work on typhoid fever has just appeared in an entirely new dress: it is already partially known to physicians in this country, through the translation of Dr. Bowditch, and the extracts and notices of it which have appeared in different journals. The body of the work,—that is, the greater part of the cases, and the description of the lesions and symptoms, is scarcely altered; but considerable additions have been made to that portion in which the diagnosis of the disease is treated of, and especially to the treatment. Of the latter portion we insert the interesting remarks on purgatives. As to the diagnosis, the author admits the diagnostic characters pointed out some years since by one of the editors of the Examiner between the fever of Paris and the peculiar form constituting the endemic typhus of the British Islands. The typhoid fever of Dr. Louis exists to a greater or less extent as a sporadic disease throughout the world, at least this is ascertained so far as authentic reports have been received, while the typhus of Great Britain is much more frequently epidemic.

The subject of contagion, which was overlooked in the former edition, is treated of at length in the present one. The author not only admits it as regards typhus, but also typhoid fever. The latter, however, certainly presents the characters of contagion to a much less degree than the former, except in certain districts, and those are chiefly country places. In cities the contagious power hardly exists, while that of typhus is evidently increased by large collections of men.

*Evacuants*—Purgatives.*

The winning enthusiasm, and the incontestible literary talent of M. Broussais, for a time rallied around him nearly all minds, all those who, from a laborious life, are not permitted to read with undiminished attention the works of celebrated men. Physicians who have some leisure—those who admit nothing without proof—still felt themselves more or less carried away by the prevailing opinion; all sought to verify, at the bedside of the sick, what was announced to them with so much warmth, even while they received with distrust propositions which did not seem demonstrated to them; and an explanation ensues from this, how it was, that at the time that I made my observations at the Hôpital de la Charité; no other recourse was had, in the treatment of what were still called fevers, but to two orders of agents—blood-letting and tonics—and how also evacuants, so highly recommended in the last century, were entirely neglected.

The neglect of purgatives at that time can be explained by other reasons, and especially by the state of imperfection in which the physicians who boasted most of their use, in the treatment of fevers, had left the history of these diseases. Until lately, in fact, we were ignorant both of their anatomical character and their truly characteristic symptoms. The errors of diagnosis must, from that circumstance alone, be frequent; and in having recourse to evacuants, they were addressing themselves to means employed in these badly determined cases, and of the efficacy of which they should therefore be very doubtful. Besides, the physicians of the last century did not agree among themselves as to the utility of emetics and purgatives in what they called fevers; another motive for not having recourse to these medicines. Suppose we add that it would have been impossible, leaving the diagnosis out, to know, by the attentive perusal of the works of the physicians of whom we speak, who was wrong, who was right, from want of materials, of numerous facts, gathered by them without distinction, without choice, exposed and analyzed in a rigorous manner; so that if the value of evacuants in the treatment of typhoid fever is put beyond doubt for a day, the honour of it

* We must apologize to our readers for the gallicisms contained in the translation; it is correctly executed by a professional friend, not much accustomed to a task of this kind. The comparison of the new French doses with the common standard, may be made by referring to one of the last numbers of the Examiner.

should belong entirely to the moderns. In the meantime, and notwithstanding the eclat of the doctrine of Broussais, some physicians, among whom we must count Lerminier at Paris, and especially M. Bretonneau at Tours, prescribed more or less frequently purgatives in the treatment of typhoid fever. But it is to a physician of the Hôpital Neckar, M. De Larroque, that the credit is due of having brought back the attention of physicians to the employment of these agents in the treatment of the disease of which we are treating.

M. De Larroque, whose ideas and precepts I shall first fully exhibit, bases the necessity of the evacuant treatment on the existence, at the commencement of typhoid fever, of a *suburral state* of the primæ viæ, to which he attributes all of the typhoid symptoms, even the alteration of the elliptical patches of Peyer, or of the follicles of Brunner, which he considers, for this reason, secondary. "It is," says he, "the action of the altered liquids on the alimentary canal which produces the alteration of these organs; it is also especially in the place where these liquids are accumulated that the organic alterations are met with; and as the corruption of these fluids is proportioned to the time the disease has lasted; as, notwithstanding the spontaneous evacuations, they remain a long time in the small intestines; as, in fine, their stagnation could not be prolonged without being introduced in a greater or less quantity into the circulation; it undergoes this absorption, and we thus can render an account of the difficulties which sooner or later are manifested in the *ensemble* of the organism."

Undoubtedly this theory does not bear a thorough scrutiny. On one side, as has been already remarked, the alteration of the fluids of which the author speaks, at least at the commencement of the disease, is not proved: on the other, liquids greatly altered, remain often stagnated in the intestinal canal, in the stomach or intestines, in cases of cancer, of complete or incomplete strangulation—of dysentery, with ulcerations of the mucous membrane of the colon, more or less deep, &c., without the symptoms of the typhoid disease being observed. These, and especially the more remarkable of them—the cerebral phenomena, and those which keep the organs of the senses impaired—develope themselves sometimes very near the commencement, when we cannot suppose at once an alteration of the fluids contained in the intestine, and their removal into the torrent of the circulation, etc.

Whatever there is in this theory, M. De Larroque does not employ evacuants in particular circumstances, in a certain period of the disease, when it assumes such and such a form; but in all its forms, during its whole course, until complete convalescence, until the fever has entirely ceased.

And purgatives are not the only evacuants to which he has recourse; he gives almost always, at the beginning of his treatment, from 5 to 6 centigrammes of tartar emetic in a large quantity of water; more rarely ipecac. on account of the nausea which it excites in many patients, and also because it brings away more rarely alvine evacuations. Given from the commencement, (p. 119,) and after a very precise indication, (that of gastric symptoms,) the emetic has, according to M. De Larroque, the advantage of sensibly shortening the course of the typhoid fever—to impress upon it, generally, a mild character, and to prevent a host of anomalies, which are developed with an intensity relative to the multiplicity and the gravity of the preceding symptoms. Never, says he, do the patients find themselves more relieved than when the emetic brings on at the same time, or successively, evacuations by the mouth, or inferiorly; and it frequently happens that patients who have their mouths dry and arid, find them become moist when copious vomiting has been induced, (p. 120.) Also, when the effect of the first emetic was inappreciable or slight, it is necessary to give it again, and in a larger dose, especially if the saburral symptoms call for the employment of such means, (121-122.)

Far from being stopped in the administration of purgatives by diarrhœa, the pains in the abdomen, and the meteorism,—these, on the contrary, are the reasons which indicate to M. De Larroque not to deviate from the employment of these medicines, (p. 125;) for in his opinion, the more the material cause of the disease is suffered to remain on the intestinal mucous membrane, the more is there reason to fear that there may be a serious alteration, which may lead to the more or less rapid death of the patient.

It will, perhaps, be objected, continues he, that when there is spontaneous diarrhœa, there is no use of administering purgatives, as nature herself procures the evacuations which I regard as necessary. But, according to the author of the memoir, nature rejects much less generally the material cause of the disease, than the liquids secreted by the inflamed surface; and then, when it takes this duty upon itself, it discharges it too slowly.

Besides, the salutary modifications produced by evacuants are much more evident usually, according to M. De Larroque, (p. 127,) according as the dejections have been more abundant, according to the haste with which we have produced them, and the little interruption in the administration of laxatives. If the dejections are stopped after having been abundantly brought on, it is rare, in the commencement of the disease, that the typhoid symptoms, which had diminished, are not reproduced, and in an aggravated form, in proportion to the negligence in again freeing the abdomen; but if we hasten to fulfil this indication, the accidents of relapse are not generally of long duration.

When, however, the patients experience colics and superpurgation, it is right to suspend, for twenty-four hours, the administration of the laxatives, because, by insisting on their use, we may add to the dothinenteric inflammation, an erythematous phlegmasia.

We can, as fast as the general state improves, interrupt from time to time the evacuations per anum; but we must not, as a general rule, cease to employ them until the disease is completely subdued, (p. 128.)

Laxatives suffice generally, and we should not have recourse to drastics, except in cases of obstinate constipation.

To the laxatives, (Seidlitz water, castor oil, calomel,) M. De Larroque joins emollient cataplasms on the abdomen, when the intestine appears very painful, acidulated drinks, emollient injections morning and evening; and he does not deviate from this treatment as long as the typhoid phenomena persist; afterwards, the fever having disappeared, he hastens the return of strength by light tonics.

The results of this treatment are very satisfactory, if we judge of them by the facts collected by M. De Larroque, and placed by him before the eyes of the Royal Academy of Medicine. In fact, the minimum duration of the treatment would be, according to these same facts, ten days, to commence with the admission of the patients into the hospital, and the mortality of a tenth only, or of ten subjects in a hundred, including those who came in dying; another circumstance which would permit the mortality to be still farther reduced, since, when we judge of the degree of influence of any therapeutic method whatever on the issue of a disease, we ought to separate from the analyzed facts those which relate to patients who have not been treated any time, or who have been treated only at a time when it was not possible to hope for success from any therapeutic agent.

I would add, that in reading, with care, the observations consigned by M. De Larroque in his memoir, we there recognise so many examples of the typhoid affection—and if all the facts comprised in his summary are as well characterized as these last—if, as there is every reason to believe, M. De Larroque has not made a selection—if he has analysed without distinction all the facts which have been presented to his observation, in a given time, it becomes extremely probable that the evacuant treatment is preferable to those which have been employed lately, better even than the one which I had first advised, *provisionally*, the results of which I have laid down at the end of the preceding chapter.

It is, besides, the more probable, as the facts analyzed by M. De Larroque are so many cases of the typhoid affection; and in no place does this physician announce that he had cut short the disease; he merely says that he abridges the course, and renders the complications less serious.

Several physicians, after M. De Larroque, have treated the disease by evacuants, with a success not less marked. I will cite among them Dr. Weber of Mulhousens, whose talents equal his scientific integrity, but who has not published, at least so I believe, the analysis of his labours on this subject; so that I cannot rigorously appreciate them.

Other practitioners, it is true, have not been so fortunate. Thus of a hundred and thirty-four patients treated by M. Piédagnel at the Hôtel Dieu, by means of evacuants, in the years 1834 and '35, nineteen died, or a seventh and a fraction. We can believe, nevertheless, from the terms of his communication made to the Royal Academy of Medicine, that M. Piédagnel has comprised among his patients some who had not the typhoid disease, and who served to augment a little the mortality indicated; so that the difference between the results obtained by him and M. De Larroque is, in reality, less than it at first seems. In fine, to appreciate better the influence of purgatives in the treatment of typhoid fever, M. Piédagnel distinguishes several varieties of this disease.

1. Simple typhoid fever, all of the cases of which, or sixty-nine in the hundred and thirty-four whom he treated, recovered.

2. Adynamic fever, which variety furnished forty-nine cases, of which ten died.

3. Ataxic typhoid fever, characterized by cerebral symptoms, and by lesions which have their seat in the encephalic organs; which furnished fourteen cases, seven of whom died.

4. Finally, severe typhoid fever, of which there were two cases, both of them fatal, and in subjects at the autopsy of whom no anatomical lesion was found.

These two last cases cannot evidently be counted in the sum of the mortality; for we cannot give the name of typhoid fever to a disease which carries off immediately a patient, without leaving traces behind; this disease might very well be an eruptive fever, or some other disease than the typhoid fever. Can we not, perhaps, say the same of several cases of the variety to which M. Piédagnel gives the name of ataxic typhoid, and the anatomical character of which would be in the brain; since, on one side, the anatomical character of typhoid fever has not its seat in the brain; and, on the other part, it is not said, if, in these same cases, the elliptical patches of Peyer were more or less deeply altered; so that the facts collected by M. De Larroque, and those by M. Piédagnel, are not entirely comparable, and the difference indicated in the results of the treatment of these two physicians, is perhaps more apparent than real, as I mentioned just now.

Of forty-eight patients treated by means of purgatives by M. Andral, at the Hospital La

Charité, and of which it is doubtful in his report to the Royal Academy of Medicine, on the subject of the work of M. De Larroque, eight, or one-sixth part died; a proportion still somewhat more considerable than that indicated by M. Piédagnel, but about which there can be no discussion, from want of details of the facts.

For my part, also, I have endeavoured to learn, by clinical facts, the value of purgatives, in the treatment of typhoid fever; and I have treated by them thirty-eight patients who came into my division, at the hospital La Pitié, in 1835, from the month of April to the month of December of the same year. The history of these cases has been collected with great care by Dr. Barth, then interne of my division.

He made a detailed analysis of them, which was published in the number of the 4th January, by the Presse Médicale; and it is from that analysis, in which no important detail has been omitted, that I extract what follows. Of the thirty-eight treated by evacuants, seven among those who recovered offered some doubt as to the character of their disease, and they have been left out of this analysis. Of the thirty-one subjects remaining and offering all the symptoms of typhoid fever, three only died, or about the tenth part. These subjects did not all have the disease in the same degree: grave with some, the disease was of moderate intensity, or light with the others.

1. The grave cases were nine in number, and offered, generally examined, to a high degree, all the symptoms of typhoid fever: prostration, stupor, cephalalgia, humming in the ears, sometimes complete deafness, giddiness, frequent stools, sometimes involuntary rose-coloured spots, numerous sudamina, meteorism of the abdomen, tongue dry, sometimes drowsiness, frequently delirium, hot skin, accelerated pulse, (from a hundred and eight to a hundred and twelve pulsations a minute.) None of the subjects of this series, or of the following, offered the agitation or furious delirium which characterized the ataxic form.

The average age was twenty-two years and a half; six were below, three above; the extremes were eighteen and forty years. Seven among them were discharged cured; two died, a little less than a fourth.

Among those who got well, the disease had attacked them on an average eleven days and a half from their entrance; less than ten days with three, more than twelve with fourteen. Their convalescence, counted from the day on which they ate the eighth of a portion, took place on an average, twenty-three days and a half after their admission into the hospital, thirty-four days and a half after the invasion of the disease; and the total duration of the disease from the commencement to the discharge of the patients from the hospital was of an average of two months.

The Seidlitz water was ordinarily given in the dose of a bottle a day, sometimes of a half bottle, or of only a glass, in the cases where the first dose was followed by six or eight stools. The use of it was instantly suspended among some patients, whose alvine evacuations were too numerous and too fatiguing; but the use of this medicine was generally continued, as recommended by M. De Larroque, until the symptoms were evidently improved; and in the last days of its administration the dose was diminished; or, without determining the dose, the patients were directed to take it until they had two or three evacuations; and in two cases when the Seidlitz water was taken with much repugnance, it was for the time replaced by castor oil given for two or three days. The ordinary drink was a solution of syrup of gum, or of the tartaric acid syrup. Three patients who had wakefulness, some delirium at night, took, with marked benefit, syrup of white poppy in a successively increasing dose, from four to ten "grammes." An infusion of bark was administered, with advantage, to a woman towards the end of the disease; and the dried extract of bark in increasing doses from five to fifteen "décigrammes" a day, produced good effects on two men whose strength was slow in returning, and who presented a general debility with absence of heat, tympanitis, and slowness of pulse.

The two patients who died were aged nineteen and twenty-four years; they were admitted into the hospital the fifth and the eighth days after the commencement of the disease; and their death took place ten and twenty-one days later, eighteen and twenty-six days after the invasion.

2. The milder cases, to the number of eight, (six men and two women,) offered also the greater part of the morbid phenomena of typhoid fever. There were, as in the preceding series, but to a less degree, malaise, shivering, cephalalgia, aching, at the beginning, after-afterwards depression of strength, more or less marked immobility of the features, epistaxis, buzzing vertigo, diarrhœa, swelling of the belly, rose-coloured lenticular spots, sudamina, generally less numerous than in the preceding series, heat, acceleration of the pulse, which beat from eighty to a hundred and eight, &c. &c. With several, the tongue was dry; with two, the pulse increased to a hundred and sixteen and a hundred and twenty-eight. One woman had involuntary stools; one patient was without diarrhœa on his entrance into the hospital; another had constipation during the two first days. In fine, one of the two women offered, on her arrival, the symptoms of an angina, with dysphagia and intense dyspnœa, for which she was bled, and had twenty-five leeches applied to the anges of the lower maxillary bones.

The average age of these patients was twenty-four years and a half; three were below. The extremes were fifteen and thirty-six. One of them died after sixty-three days' duration of the disease, and forty-eight days after his entrance into the hospital, in which all the signs of phthisis developed themselves; and on opening the body, tubercles were found in both lungs, with cavities on the left side; the elliptical patches of Peyer were ulcerated, and in part cicatrized. He had had no bloody discharges, and the Seidlitz water had been given during seven days from the day after his admission into the hospital. In the seven other patients the commencement of the disease took place on an average seven days and a half before their entrace into La Pitié, and two only were admitted after the tenth day. The convalescence took place twenty days after the appearance of the first symptoms,—and the total duration of the disease, from the first symptoms to the discharge of the patients, was thirty-two days.

The Seidlitz water was administered to these patients in the same manner as it was in those of the preceding series: it was displaced for a time, by the castor oil, in two cases. The drink was lemonade, with syrup of tartaric acid. With one of the two females, the syrup of white poppy was administered for two days with benefit, and from time to time emollient injections were associated with the preceding measures.

3. The subjects in whom the disease was slight amounted to fourteen; a deduction made of those the diagnosis of whose disease had not that degree of certainty which ought, observes M. Barth, to have facts which serve as the basis of a good statistic. Two of these cases had no diarrhœa; but the group of the other symptoms which they experienced, (cephalalgia, rumbling, prostration from the first, acceleration of the pulse, rose-coloured lenticular spots, sudamina, &c.,) suffices to characterize the disease, in such a manner as to leave no doubt on that point. The average age was twenty-five years; six were below, eight above; the extremes were fifteen and thirty-five. The commencement of the disease took place on an average nine days previous to their entrance into the hospital. Ten among them were admitted before, four after this term. All left the hospital cured. (*To be continued.*)

